# A Multicenter, Randomized, Open-Label Study to Compare the Effects of Gemigliptin Add-on or Escalation of Metformin Dose on Glycemic Control and Safety in Patients with Inadequately Controlled Type 2 Diabetes Mellitus Treated with Metformin and SGLT-2 Inhibitors (SO GOOD Study)

**DOI:** 10.1155/2024/8915591

**Published:** 2024-01-05

**Authors:** Hae Jin Kim, Jung Hyun Noh, Min Kyong Moon, Sung Hee Choi, Seung-Hyun Ko, Eun-Jung Rhee, Kyu Yeon Hur, In-Kyung Jeong

**Affiliations:** ^1^Department of Endocrinology & Metabolism, Ajou University School of Medicine, Suwon, Republic of Korea; ^2^Division of Endocrinology and Metabolism, Department of Medicine, Ilsan Paik Hospital, Inje University College of Medicine, Goyang, Republic of Korea; ^3^Division of Endocrinology and Metabolism, Department of Internal Medicine, Seoul Metropolitan Government Seoul National University Boramae Medical Center, Seoul National University College of Medicine, Seoul, Republic of Korea; ^4^Division of Endocrinology and Metabolism, Department of Internal Medicine, Seoul National University Bundang Hospital, Seoul National University College of Medicine, Seoul, Republic of Korea; ^5^Division of Endocrinology and Metabolism, Department of Internal Medicine, St. Vincent's Hospital, College of Medicine, The Catholic University of Korea, Seoul, Republic of Korea; ^6^Department of Endocrinology and Metabolism, Kangbuk Samsung Hospital, Sungkyunkwan University School of Medicine, Seoul, Republic of Korea; ^7^Division of Endocrinology and Metabolism, Department of Medicine, Samsung Medical Center, Sungkyunkwan University School of Medicine, Seoul, Republic of Korea; ^8^Division of Endocrinology and Metabolism, Department of Internal Medicine, Kyung Hee University Hospital at Gangdong, Kyung Hee University School of Medicine, Seoul, Republic of Korea

## Abstract

**Background:**

We aimed to compare efficacy and safety between gemigliptin add-on and escalation of the metformin dose in patients with inadequately controlled type 2 diabetes mellitus (T2DM) despite treatment with metformin and SGLT2 inhibitors.

**Methods:**

This study was a multicenter, randomized, open-label, active-controlled, parallel-group comparative study. Patients with T2DM uncontrolled on metformin and SGLT2 inhibitors were randomized to receive gemigliptin 50 mg as an add-on (GEM group, *n* = 37) or escalation of the metformin dose (500 mg, MET group, *n* = 38) for 24 weeks. The primary endpoint was the change in glycosylated hemoglobin (HbA1c) from baseline to week 24.

**Results:**

At weeks 12 and 24, the reduction in HbA1c levels was significantly greater in the GEM group than in the MET group (GEM vs. MET = −0.64% ± 0.34% vs. −0.36% ± 0.50%, *p* = 0.009 at week 12; −0.61% ± 0.35% vs. −0.33% ± 0.70%, *p* = 0.045 at week 24). The proportions of patients who achieved target HbA1c levels of <7.0% at weeks 12 and 24 and <6.5% at week 12 were greater in the GEM group than in the MET group. An index of *β*-cell function was also significantly improved in the GEM group. The safety profiles were similar between the two groups.

**Conclusions:**

Gemigliptin add-on therapy may be more effective than metformin dose escalation in patients with T2DM insufficiently controlled using metformin and SGLT2 inhibitors, without safety concerns. This trial is registered with CRIS_number: KCT0003520.

## 1. Introduction

Effective glycemic control is required to delay or prevent the development of microvascular and macrovascular complications in patients with type 2 diabetes mellitus (T2DM) [1]. However, as the disease progresses, most patients are unlikely to achieve sustained glycemic control without treatment intensification, and combination therapy with various antidiabetic agents is often necessary [2].

Metformin is the most widely used first-line antidiabetic drug according to multiple guidelines for the treatment of T2DM [3, 4]. Sodium-glucose cotransporter 2 (SGLT2) inhibitors improve glycemic control by blocking glucose reabsorption in the proximal convoluted tubules of the kidney and increasing glycosuria. Beyond their glucose-lowering effects, SGLT2 inhibitors have been associated with beneficial effects such as weight loss and decreases in blood pressure [5]. The combination of metformin and SGLT2 inhibitors has been shown to improve glycemic control and weight loss and to reduce cardiovascular and renal risks in patients with T2DM. Given its demonstrated ability to protect cardiovascular and renal function independent of glucose control in high-risk patients, this combination therapy has been increasingly recommended, particularly for patients with T2DM who also have atherosclerotic cardiovascular disease, heart failure, or renal disease [3, 4, 6].

When glucose control remains insufficient despite combined treatment with metformin and SGLT2 inhibitors, the dose of metformin can be increased, or another class of antidiabetic medication can be added. Some patients are unable to tolerate metformin, and metformin-associated gastrointestinal adverse events (AEs) such as diarrhea and nausea may depend on metformin dosage [7]. Dipeptidyl peptidase-4 (DPP4) inhibitors are widely used antidiabetic medications that block the action of the DPP4 enzyme, which breaks down incretin hormones and improves glucose control via glucose-dependent secretion of insulin and suppression of glucagon [8]. DPP4 inhibitors are often used as second- or third-line therapies for T2DM because of their ease of use, low risk of hypoglycemia, weight neutrality, and favorable tolerability [9]. Combined treatment with DPP4 inhibitors and SGLT2 inhibitors is an attractive option given their complementary mechanisms of action and the demonstrated effectiveness and tolerability of the combination in patients with T2DM [10, 11]. Gemigliptin is a potent and selective DPP4 inhibitor that has been investigated in combination with various antidiabetic drugs, including metformin, metformin plus sulfonylureas, and insulin [12].

Triple-combination treatment with DPP4 inhibitors added to metformin plus SGLT2 inhibitors may exhibit synergism with complementary actions, thereby offering an effective therapeutic option. However, no studies have compared their effects with the uptitration of metformin, which is another option for patients with uncontrolled T2DM despite treatment with metformin and SGLT2 inhibitors. Accordingly, this study is aimed at comparing the efficacy and safety of gemigliptin add-on versus metformin dose escalation in patients with T2DM inadequately controlled using metformin and SGLT2 inhibitors.

## 2. Methods

### 2.1. Study Design and Participants

This investigator-initiated, multicenter, randomized, open-label, and active-controlled trial was conducted across eight sites in Korea from July 2019 to February 2022 (CRIS_number: KCT0003520).

We enrolled patients who met the following criteria: (a) presence of T2DM, (b) age 20–75 years, (c) body mass index (BMI) of 18.5–40 kg/m^2^, (d) poor glycemic control (7.0% ≤ HbA1c ≤ 10%), and (e) treatment with a stable dose of 1,000–2,000 mg metformin in combination with SGLT2 inhibitors (10 mg dapagliflozin, 10 mg or 25 mg empagliflozin, or 50 mg ipragliflozin) for at least 8 weeks. The key exclusion criteria were as follows: (a) history of type 1 diabetes; (b) acute metabolic complications of diabetes within the past 6 months; (c) history of cardiovascular diseases such as myocardial infarction or angina, percutaneous transluminal coronary angioplasty, or stroke within 6 months before screening; (d) history of allergy or hypersensitivity to DPP4 inhibitors; (e) use of prohibited concomitant medications (systemic corticosteroids, antiobesity drugs, cyclosporine, etc.) within the previous 4 weeks; (f) drug or alcohol abuse within 3 months prior to screening; (g) aspartate aminotransferase (AST) or alanine aminotransferase (ALT) level 3 times higher than the upper limit of the normal range; and (h) estimated glomerular filtration rate < 45 mL/min/1.73 m^2^ before screening.

Eligible participants were randomized 1 : 1 to receive gemigliptin 50 mg/day as an add-on (GEM group) or metformin 500 mg/day escalation (MET group) for 24 weeks. Computer-generated randomization was used, and allocation concealment was achieved using sealed opaque envelopes that were sequentially numbered and kept in a locked cabinet until the time of randomization. Concomitant baseline antidiabetic regimens were maintained throughout the study period. The following baseline data were collected: demographic characteristics, medical and medication histories, physical examination results, fasting plasma glucose (FPG), HbA1c, fasting insulin, lipid profiles (total cholesterol, low-density lipoprotein cholesterol (LDL-C), high-density lipoprotein cholesterol (HDL-C), and triglycerides), and laboratory test results for safety (levels of blood creatinine, AST, ALT, complete blood count, and urinalysis). The postprandial glucose (PPG) level was recorded as the average 2-hour PPG after every meal during the 3 days prior to the visit using the self-monitored blood glucose (SMBG) levels of the patients. The homeostasis model assessment of insulin resistance (HOMA-IR) ([fasting insulin (*μ*U/mL) × fasting glucose (mmol/L)]/22.5) value was calculated as an index of insulin resistance, and the HOMA of *β*-cell function (HOMA-*β*) ([20 × fasting insulin (*μ*U/mL)/fasting glucose (mmol/L)] − 3.5) value was calculated as an index of beta cell function. Follow-up visits were scheduled 12 and 24 weeks after enrollment. At each visit, body weight, vital signs, HbA1c, FPG levels, PPG levels, and AEs were assessed. Fasting insulin, lipid profiles, urine albumin-to-creatinine ratio, serum ketone bodies (acetoacetate, total ketone, and *β*-hydroxybutyric acid), and laboratory values for safety were assessed at baseline and week 24.

Patients were withdrawn during the study under one of the following conditions: drug compliance of less than 80%; HbA1c level of >10.0% at week 12; and withdrawal of consent, at the investigators' discretion, or in certain situations such as significant intercurrent illness or a serious adverse event (SAE) during the trial.

This study was conducted in accordance with the Declaration of Helsinki, and the protocol was approved by the Institutional Review Board (IRB) at each participating site, including the Kyung Hee University Hospital IRB at Gangdong (KHNMC 2018-11-002). All patients provided written informed consent before participating in the study.

### 2.2. Outcome Measures

The primary efficacy endpoint was the change in HbA1c levels from baseline to week 24. The secondary efficacy endpoints were as follows: changes in HbA1c level from baseline to week 12, proportions of participants achieving HbA1c < 6.5% and HbA1c < 7.0% at week 24, changes in FPG and PPG levels at weeks 12 and 24, changes in lipid profiles from baseline to week 24, and changes in HOMA-IR and HOMA-*β* from baseline to week 24. The exploratory endpoints were changes in the urine albumin/creatinine ratio and serum ketone bodies from baseline to week 24.

Safety was assessed by monitoring the overall incidence of AEs, vital signs, and laboratory and physical examination results. All AEs were recorded and assessed by the investigator to determine their possible relationship with the study interventions. Regarding hypoglycemia, any patient who reported an SMBG level < 70 mg/dL with or without symptoms was considered to have experienced a hypoglycemic episode.

### 2.3. Statistical Analysis

The sample size for the comparison between the MET and GEM groups was determined using a two-sample *t*-test with a targeted power of 80% and a significance level of 0.05. Assuming a treatment difference of 0.5% and a standard deviation of 0.8% [13, 14], the calculated sample size, accounting for a 10% anticipated dropout rate, was 37 per group.

Patients who completed the 24-week treatment period without major protocol deviations were included for the efficacy analyses, and all randomized patients who had been administered the study drug at least once were included for the safety analyses. Baseline demographic and biochemical parameters were summarized using descriptive statistics: continuous variables are reported as the mean and standard deviation or median and interquartile range, while categorical values are reported as counts and percentages. To analyze the efficacy endpoints at weeks 12 and 24, we used the independent *t*-test or Wilcoxon rank-sum test for continuous variables after normality tests with the Shapiro-Wilk test and the *χ*^2^ test for categorical variables. The HbA1c levels measured repeatedly from baseline to 24 weeks were analyzed using a mixed model for repeated data. Group differences at each time point were analyzed using the Bonferroni post hoc analysis for multiple comparisons. Subgroup analyses of changes in HbA1c levels were performed according to baseline characteristics. An interaction analysis of covariates that affected the 24-week reduction in HbA1c in the GEM group compared with that in the MET group (reference) was conducted using a general linear model adjusted for baseline HbA1c. Safety analyses were performed using Fisher's exact test. Statistical significance was set at *p* < 0.05. SAS9.4 (Statistical Analysis System version, SAS Institute, Cary, NC, USA) and R4.1.0 were used for statistical analyses.

## 3. Results

### 3.1. Baseline Characteristics

**Figure 1:**
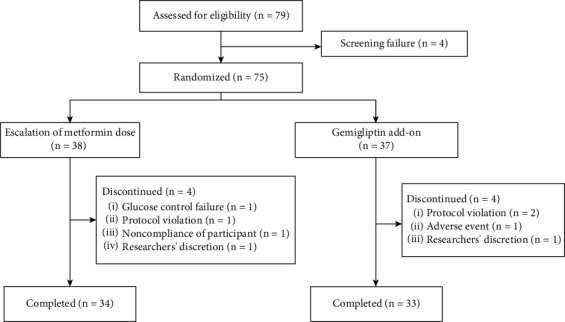
Study flowchart.

Of the 79 screened patients, 75 were randomized to receive the study medication (38 in the MET group and 37 in the GEM group), 67 of whom completed the study (Figure 1).

The baseline demographic and clinical characteristics were well balanced between the two groups (Table 1). The overall mean patient age was 52.36 ± 11.86 years, and the mean BMI was 28.06 ± 4.44 kg/m^2^. The mean duration of T2DM was 7.37 ± 4.90 years, and the mean baseline HbA1c level was 7.59 ± 0.52%. The mean dose of metformin was 1306.72 ± 431.86 mg/day. No significant differences in metabolic parameters, metformin dosage, or use of concomitant medications were observed between the two groups at baseline.

### 3.2. Efficacy

**Figure 2:**
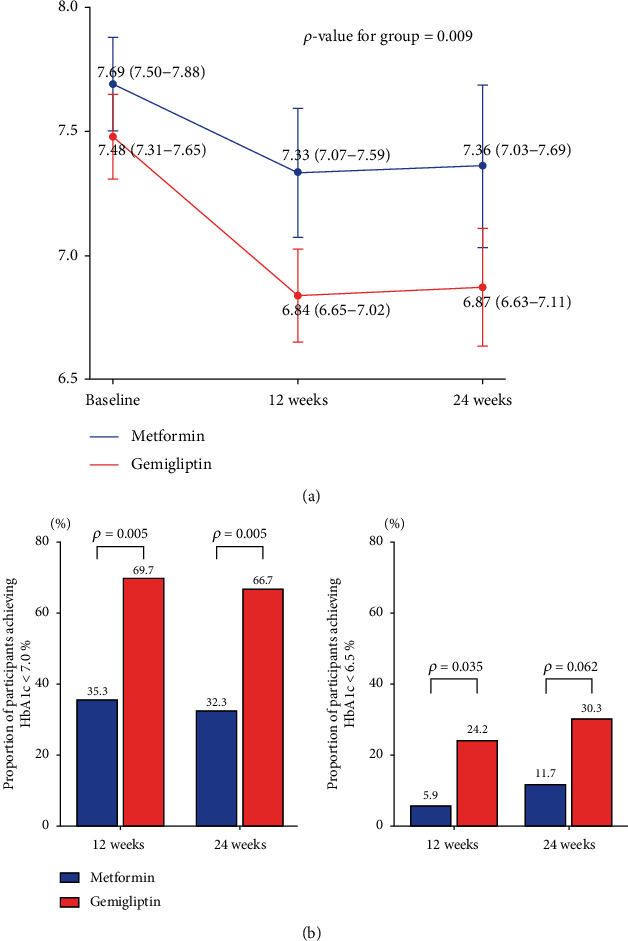
(a) Changes in HbA1c level from baseline to week 24. When HbA1c repeatedly measured from baseline to 24 weeks were analyzed using a mixed model for repeated data, significant differences were observed between the groups (*p* for group = 0.009). There was a significant difference between the two groups at 12 weeks and 24 weeks through the Bonferroni post hoc correction. Error bars indicate 95% confidence intervals. (b) Proportion of participants achieving HbA1c < 6.5% and HbA1c < 7.0% at weeks 12 and 24.

**Figure 3:**
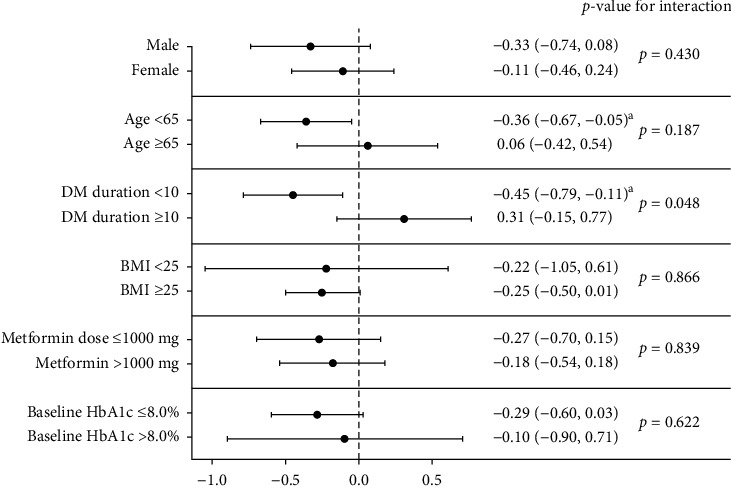
Subgroup analysis of changes in HbA1c at week 24 according to baseline characteristics. The *p* value reflects the interaction analysis of covariates affecting the 24-week reduction in HbA1c in the GEM group when compared with that in the MET group (reference). Analyses were performed using a general linear model adjusted for baseline HbA1c. Points represent the coefficient estimate, and the error bar shows the 95% confidence interval of the coefficient estimate. ^a^*p* < 0.05.

At weeks 12 and 24, the changes in HbA1c levels from baseline were significantly greater in the GEM group than in the MET group (GEM vs. MET = −0.64% ± 0.34% vs. −0.36% ± 0.50%, *p* = 0.009 at week 12; −0.61% ± 0.35% vs. −0.33% ± 0.70%, *p* = 0.045 at week 24; Table 2). When HbA1c repeatedly measured from baseline to 24 weeks were analyzed using a mixed model for repeated data, significant differences were observed between the groups (*p* for group = 0.009, Figure 2(a)). There was a significant difference between two groups at 12 weeks and 24 weeks through the Bonferroni post hoc correction, a multiple comparison test. The proportion of patients who achieved a target HbA1c level of <7.0% at weeks 12 and 24 was greater in the GEM group than in the MET group (GEM vs. MET = 69.7% vs. 35.3%, *p* = 0.005 at week 12; 66.7% vs. 32.3%, *p* = 0.005 at week 24; Figure 2(b)). The proportion of patients achieving a target HbA1c level of <6.5% at week 12 was also greater in the GEM group (24.2% vs. 5.9%, *p* = 0.035; Figure 2(b)). At week 24, the HOMA-*β* value had significantly improved from baseline in the GEM group (GEM vs. MET = 15.60% ± 30.36 vs. −4.48% ± 32.57, *p* = 0.011; Table 2). The changes in FPG and PPG levels from baseline to weeks 12 and 24 were comparable between the groups, as were the changes in lipid profile from baseline to week 24. In the subgroup analyses according to baseline characteristics, a greater reduction in HbA1c levels was observed in the GEM group than in the MET group among patients aged <65 years (-0.36 [CI: -0.67, -0.05]) and those with a T2DM duration < 10 years (-0.45 [CI: -0.79, -0.11]) (Figure 3).

No significant differences in the changes in the UACR or serum ketone bodies were observed (Table 2).

### 3.3. Safety

The safety results are summarized in Table 3. There were no SAEs during the study period, and one patient in the GEM group withdrew owing to an AE (urticaria). The AE profiles were similar between the two groups. Hypoglycemia or genital infection was not observed in either group.

## 4. Discussion

The present results highlight gemigliptin add-on therapy as an effective treatment of choice compared with escalation of metformin dose in patients with inadequately controlled T2DM despite treatment with metformin and SGLT2 inhibitors, and no significant safety issues were noted. Moreover, the addition of gemigliptin to metformin and SGLT2 inhibitors was shown to improve *β*-cell function.

T2DM is a multifactorial and progressive disease, and its pathogenesis involves multiple mechanisms. Therefore, combination therapy using various antidiabetic agents with complementary modes of action is recommended [2]. Combined treatment with DPP4 and SGLT2 inhibitors has been proposed as an effective treatment option for T2DM given their complementary mechanisms of action and low risk of hypoglycemia or weight gain [10, 11, 15]. SGLT2 inhibitors increase endogenous glucose production; however, the addition of a DPP4 inhibitor, which inhibits glucose production, can compensate for this increase [16]. In addition, meta-analyses of DPP4 inhibitors or SGLT2 inhibitors have suggested that these drugs exert beneficial effects on glycemic variability [17, 18], and one randomized study reported that SGLT2 inhibitors combined with DPP4 inhibitor therapy strongly reduced glycemic fluctuations when compared with SGLT2 inhibitor monotherapy [19]. Moreover, cardiovascular outcome trials have demonstrated the cardiovascular safety of DPP4 inhibitors and the cardiovascular benefits of SGLT2 inhibitors [20]. In another meta-analysis, combined treatment with SGLT2 inhibitors and DPP4 inhibitors enhanced effects on HbA1c reduction (−0.47; 95% CI, −0.58 to −0.37%), exerted a neutral effect on weight (0.19; 95% CI, −0.11 to 0.48 kg), and attenuated the risk of genital infections (0.73; 95% CI, 0.54 to 0.97) versus treatment with SGLT2 inhibitors alone [21]. Triple-combination therapy with metformin, DPP4 inhibitors, and SGLT2 inhibitors can target multiple pathophysiological pathways for T2DM, affecting at least six of eight components in the “ominous octet” [22], and appears to strike an appropriate balance among efficacy, safety, and tolerability profiles. In a previous randomized study of triple-combination therapy with metformin, DPP4 inhibitors, and SGLT2 inhibitors, the authors reported a significantly greater reduction in HbA1c at 24 weeks with saxagliptin add-on versus placebo add-on in patients already taking dapagliflozin plus metformin (difference, −0.35% [95% CI −0.52% to −0.18%]) [23].

In the current study, we compared the effects of gemigliptin with escalation of the metformin dose rather than with those of placebo. A previous meta-analysis demonstrated that an increase in metformin dose resulted in a further modest reduction in HbA1c of 0.26% in trials comparing lower doses with higher doses, up to a metformin dose of 2,000 mg [14]. Another previous randomized study compared the efficacy of combined treatment with the DPP4 inhibitor and metformin with metformin uptitration in Chinese patients with T2DM inadequately controlled with metformin monotherapy. The results indicated that combination therapy with vildagliptin and metformin was more effective in reducing HbA1c levels than metformin uptitration at week 24 (−0.54% vs. −0.40%, difference, 0.15% [95% CI −0.22% −0.07%]) [24]. In the present study, the HbA1c reduction at week 24 was −0.61 ± 0.35% in the GEM group and −0.33 ± 0.70% in the MET group, suggesting a greater hypoglycemic effect of gemigliptin add-on than metformin uptitration. The present study is the first to demonstrate that triple-combination treatment with DPP4 inhibitors, SGTL2 inhibitors, and metformin exerts a better hypoglycemic effect and more effectively protects beta cells than increasing the dose of metformin among patients with T2DM with inadequately controlled disease despite treatment with metformin and SGLT2 inhibitors.

Our results also revealed improvements in HOMA-*β* as well as HbA1c following the addition of gemigliptin, in accordance with previous findings. In a double-blind randomized controlled trial, initial combination therapy with gemigliptin and metformin produced improvements in HOMA-*β* values when compared with metformin monotherapy [25]. A meta-analysis also reported that gemigliptin was superior to placebo in terms of the effects on HbA1c, FPG, and HOMA-*β* [26]. In a study examining the effects of DPP4 and/or SGLT2 inhibitors in the early and advanced phases of diabetes in *db/db* mice, the authors observed that the combination of DPP4 and SGLT2 inhibitors exerted greater beneficial effects on *β*-cell mass and function, especially in the early phase of diabetes rather than an advanced phase [27]. In our subgroup analysis, a reduction in HbA1c levels was significantly greater in the GEM group than in the MET group among patients with younger age or shorter duration of T2DM. Given these findings, intensive combination treatment should be initiated early to prevent the progression of *β*-cell failure.

Several studies have demonstrated the beneficial effects of DPP4 inhibitors on lipid profiles, and gemigliptin has been reported to slightly decrease total cholesterol, LDL-C, and triglyceride levels [12]. In the present study, the GEM group exhibited a tendency towards decreased triglyceride levels, although the change was not statistically significant.

There were no significant differences in the incidence of AEs between the two groups in the present study. There were no hypoglycemic events in either group, which is in accordance with the low risk of hypoglycemia noted for metformin, SGTL2 inhibitors, and DPP4 inhibitors. One adverse drug reaction (urticaria) was observed in the GEM group, which is a well-recognized AE associated with DPP4 inhibitors [28]. In this study, there were no gastrointestinal AEs in the MET group, which may be attributed to the fact that the patients were not metformin-naïve before enrollment and underwent gradual dose escalation [7]. Further, no genitourinary tract infections occurred in either group.

This study had some limitations, including the relatively small number of participants. Because we calculated the sample size based on the primary outcome before the trial, some secondary efficacy outcomes, such as changes in FPG and PPG levels, may not have included enough participants to reveal statistically significant differences. Second, although there was a greater reduction in HbA1c levels in the GEM group, there were no significant differences in FPG or PPG levels between the groups, which may be because 2-hour PPG levels were only examined across 3 days. More precise methods of glucose evaluation, such as 7-point SMBG or continuous glucose monitoring over a longer duration, would have provided a more detailed glucose profile.

## 5. Conclusions

In conclusion, the current results highlight gemigliptin add-on therapy as an effective treatment option when compared with metformin dose escalation in patients with T2DM exhibiting inadequate glycemic control using metformin and SGLT2 inhibitors, without safety concerns.

## Figures and Tables

**Table 1:** Baseline characteristics of the included participants.

	Metformin (*n* = 34)	Gemigliptin (*n* = 33)	*p* value
Age (y)	54.12 ± 12.68	50.55 ± 10.85	0.220
Male, *n* (%)	20 (58.82)	21 (63.64)	0.686
DM duration (y)	8.09 ± 5.34	6.64 ± 4.36	0.229
Weight (kg)	77.29 ± 14.88	78.37 ± 14.30	0.762
BMI (kg/m^2^)	28.23 ± 4.61	27.89 ± 4.33	0.760
Waist circumference (cm)	95.44 ± 11.61	96.26 ± 12.95	0.786
Hypertension, *n* (%)	23 (67.65)	18 (54.55)	0.271
Cardiovascular disease, *n* (%)	4 (11.76)	6 (18.18)	0.461
Dyslipidemia, *n* (%)	28 (82.35)	24 (72.73)	0.345
Systolic blood pressure (mmHg)	128.0 ± 9.5	126.8 ± 10.9	0.613
Diastolic blood pressure (mmHg)	78.0 ± 8.7	78.5 ± 8.3	0.839
HbA1c (%)	7.69 ± 0.54	7.48 ± 0.49	0.095
Fasting plasma glucose (mg/dL)	136.82 ± 20.83	140.73 ± 20.33	0.441
Postprandial glucose (mg/dL)	190.06 ± 35.81	191.59 ± 44.32	0.879
Fasting insulin (mIU/L)	10.46 ± 7.07	8.47 ± 5.02	0.191
HOMA-IR	3.52 ± 2.41	2.97 ± 1.92	0.306
HOMA-*β*	57.13 ± 44.22	41.03 ± 22.80	0.066
Total cholesterol (mg/dL)	151.47 ± 33.30	154.92 ± 31.36	0.665
Triglyceride (mg/dL)	149.50 ± 75.51	157.30 ± 85.57	0.693
HDL-C (mg/dL)	49.32 ± 12.33	51.24 ± 12.34	0.527
LDL-C (mg/dL)	75.70 ± 23	76.67 ± 28.36	0.879
eGFR (mL/min/1.73 m^2^)	99.61 ± 21.43	96.58 ± 17.06	0.501
AST (U/L)	26.26 ± 9.74	30.12 ± 21.52	0.352
ALT (U/L)	31.88 ± 16.73	32.76 ± 20.55	0.849
UACR	17.8 (34.9)	511.7 (13.7)	0.171
Acetoacetate (*μ*mol/L)	102.1 (138.2)	121.7 (183.6)	0.367
Total ketone (*μ*mol/L)	287.3 (254.8)	315.2 (365.7)	0.407
*β*-Hydroxybutyric acid (*μ*mol/L)	143.2 (171.2)	197.1 (203.3)	0.533
Antidiabetic medication prior to randomization			
SGLT2 inhibitor			
Dapagliflozin, *n* (%)	15 (44.12)	17 (51.52)	0.801
Empagliflozin, *n* (%)	15 (44.12)	12 (36.36)	
Ipragliflozin, *n* (%)	4 (11.76)	4 (12.12)	
Metformin dosage (mg/day)	1285.29 ± 441.16	1328.79 ± 427.75	0.684
Concomitant medications			
Antihypertensive drugs, *n* (%)	20 (58.82)	16 (48.48)	0.396
Antidyslipidemic drugs, *n* (%)	30 (88.24)	26 (78.79)	0.297
Antiplatelet drugs, *n* (%)	9 (26.47)	5 (15.15)	0.255

Values are presented as mean ± standard deviation, median (interquartile range), or number (%). BMI: body mass index; HOMA-IR: homeostasis model assessment of insulin resistance; HOMA-*β*: homeostasis model assessment of *β*-cell function; HDL-C: high-density lipoprotein cholesterol; LDL-C: low-density lipoprotein cholesterol; AST: aspartate aminotransferase; ALT: alanine aminotransferase; UACR: urine albumin-to-creatinine ratio.

**Table 2:** Study outcomes according to treatment group.

	Metformin (*n* = 34)	Gemigliptin (*n* = 33)	*p* value
HbA1c (%)			
Baseline	7.69 ± 0.54	7.48 ± 0.49	0.095
At week 12	7.33 ± 0.75	6.84 ± 0.53	0.003
At week 24	7.36 ± 0.94	6.87 ± 0.67	0.017
Change from baseline at week 12	−0.36 ± 0.50	−0.64 ± 0.34	0.009
Change from baseline at week 24	−0.33 ± 0.70	−0.61 ± 0.35	0.045
Fasting plasma glucose (mg/dL)			
Baseline	136.82 ± 20.83	140.73 ± 20.33	0.441
At week 12	130.94 ± 22.23	132.48 ± 30.40	0.813
At week 24	135.35 ± 30.68	128.76 ± 18.97	0.293
Change from baseline at week 12	−5.88 ± 19.56	−8.24 ± 23.62	0.657
Change from baseline at week 24	−1.47 ± 30.07	−11.97 ± 17.89	0.087
Postprandial glucose (mg/dL)			
Baseline	190.06 ± 35.81	191.59 ± 44.32	0.879
At week 12	175.22 ± 43.30	166.18 ± 33.69	0.368
At week 24	170.89 ± 35.49	168.71 ± 39.13	0.822
Change from baseline at week 12	−15.31 ± 31.39	−22.11 ± 21.73	0.345
Change from baseline at week 24	−19.55 ± 32.88	−22.97 ± 29.27	0.680
Fasting insulin (mIU/L)			
Baseline	10.46 ± 7.07	8.47 ± 5.02	0.191
At week 24	9.57 ± 4.60	9.99 ± 7.19	0.781
Change from baseline at week 24	−0.88 ± 5.43	1.52 ± 5.42	0.075
HOMA-IR			
Baseline	3.52 ± 2.41	2.97 ± 1.92	0.306
At week 24	3.21 ± 1.66	3.24 ± 2.42	0.957
Change from baseline at week 24	−0.31 ± 1.99	0.27 ± 1.98	0.238
HOMA-*β*			
Baseline	57.13 ± 44.22	41.03 ± 22.80	0.066
At week 24	52.65 ± 28.99	56.63 ± 41.18	0.649
Change from baseline at week 24	−4.48 ± 32.57	15.60 ± 30.36	0.011
Total cholesterol (mg/dL)			
Baseline	151.47 ± 33.30	154.92 ± 31.36	0.665
At week 24	144.31 ± 31.37	147.78 ± 29.36	0.642
Change from baseline at week 24	−7.16 ± 22.69	-7.14 ± 18.70	0.996
Triglyceride (mg/dL)			
Baseline	149.50 ± 75.51	157.30 ± 85.57	0.693
At week 24	145.35 ± 81.15	136.09 ± 74.40	0.628
Change from baseline at week 24	−4.15 ± 60.36	−21.21 ± 55.71	0.234
HDL-C (mg/dL)			
Baseline	49.32 ± 12.33	51.24 ± 12.34	0.527
At week 24	49.21 ± 13.04	51.06 ± 11.13	0.534
Change from baseline at week 24	−0.12 ± 5.97	−0.18 ± 7.54	0.969
LDL-C (mg/dL)			
Baseline	75.70 ± 23.00	76.67 ± 28.36	0.879
At week 24	67.12 ± 25.87	71.65 ± 24.16	0.462
Change from baseline at week 24	−8.58 ± 18.08	−5.02 ± 18.13	0.424
UACR			
Baseline	17.8 (34.9)	11.7 (13.7)	0.171
At week 24	19.4 (48.3)	12.8 (10.0)	0.107
Change from baseline at week 24	0.4 (15.7)	-0.3 (12.7)	0.541
Acetoacetate (*μ*mol/L)			
Baseline	102.1 (138.2)	121.7 (183.6)	0.367
At week 24	86.2 (120.2)	90 (152.0)	0.779
Change from baseline at week 24	-18.4 (159.8)	-2.4 (244.6)	0.817
Total ketone (*μ*mol/L)			
Baseline	287.3 (254.8)	315.2 (365.7)	0.407
At week 24	231.4 (212.1)	205.2 (282.2)	0.827
Change from baseline at week 24	-38.0 (310.6)	-20.0 (485.7)	0.640
*β*-Hydroxybutyric acid (*μ*mol/L)			
Baseline	143.2 (171.2)	197.1 (203.3)	0.533
At week 24	121.9 (114.6)	114.6 (109.8)	0.856
Change from baseline at week 24	-37.0 (166.4)	-24.4 (242.8)	0.764

Values are presented as mean ± standard deviation or median (interquartile range). *p* values were applied by independent *t*-test or the Wilcoxon rank-sum test. HOMA-IR: homeostasis model assessment of insulin resistance; HOMA-*β*: homeostasis model assessment of *β*-cell function; HDL-C: high-density lipoprotein cholesterol; LDL-C: low-density lipoprotein cholesterol; UACR: urine albumin-to-creatinine ratio.

**Table 3:** Adverse events.

	Metformin (*n* = 38)	Gemigliptin (*n* = 37)
Serious adverse events	0	0
Drug withdrawn due to adverse event	0	1 (2.70)
Adverse events		
Hypoglycemia	0	0
Pancreatitis	0	0
Genital infection	0	0
Urinary tract infection	0	0
Liver enzyme elevation^a^	0	0
Creatinine elevation^b^	1 (2.63)	0
Leukocytosis^c^	0	0
Urticaria	0	1 (2.70)
Others		
Dim eyes	1 (2.63)	0
Diabetic retinopathy	1 (2.63)	0
Diabetic neuropathy	0	1 (2.70)
Herpes zoster	0	1 (2.70)
Colon polyps	1 (2.63)	0

Values are presented as number (%). ^a^AST or ALT 3 times higher than the upper limit of the normal range. ^b^Creatinine > 1.4 mg/dL. ^c^White blood cell count > 10,000/*μ*L. AST: aspartate aminotransferase; ALT: alanine aminotransferase.

## Data Availability

The data used to support the findings of this study are available from the corresponding author upon reasonable request.
